# Effects of Tanreqing injection on the gut microbiota in healthy volunteers

**DOI:** 10.3389/fcimb.2024.1428476

**Published:** 2024-10-04

**Authors:** Shiyu Li, Wenxia Zhang, Sijie Liu, Yichen Zhou, Wei Liu, Weian Yuan, Min He

**Affiliations:** ^1^ Clinical Research Center, Shuguang Hospital Affiliated to Shanghai University of Traditional Chinese Medicine, Shanghai, China; ^2^ Department of Clinical Laboratory, Zhoupu Hospital, Shanghai University of Medicine and Health Sciences, Shanghai, China; ^3^ Department of Pharmacy, The State Administration of Traditional Chinese Medicine (SATCM) Third Grade Laboratory of Traditional Chinese Medicine Preparations, Shuguang Hospital Affiliated to Shanghai University of Traditional Chinese Medicine, Shanghai, China

**Keywords:** Tanreqing injection, gut microbiota, microbial diversity, healthy volunteers, TCM

## Abstract

**Objectives:**

Many studies have confirmed that antibacterial agents can disrupt the human gut microbiota. In China, Tanreqing injection (TRQ) is a drug with antibacterial activity that is widely used in the treatment of respiratory infections. However, its specific influence on gut microbiota remains unclear. This study aimed to investigate the effect of TRQ on the gut microbiota of healthy volunteers.

**Methods:**

Twelve healthy adults received 20 ml of TRQ intravenously daily for 7 consecutive days. At six timepoints (Pre, on D1, D3, D5, D7 and follow-up visit) fecal samples were collected and analyzed using 16S rRNA gene sequencing.

**Results:**

Eleven people were included in the analysis finally. TRQ did not significantly alter gut microbiota diversity or richness (Shannon and Simpson and Chao1 index) in healthy people during the intervention. Gut microbial structure was stable (weighted and unweighted Unifrac). Using a machine learning method based on PLS-DA analysis, the separation trend on D7 at the genus level was found, returning to baseline two days after discontinuation. The abundance of major genus fluctuated on D7 compared with that prior to treatment, including an increase of unclassified_f_Enterobacteriaceae (13.0611%), a decrease of *Bifidobacterium* and *Escherichia-Shigella* (6.887%, 10.487%). Functional prediction analysis did not reveal any significant difference.

**Conclusions:**

Our study showed short-term use of TRQ at conventional doses may not cause perturbations to the gut microbiota in healthy adults. This finding provides some useful information for the safe use of TRQ in the treatment of respiratory infections.

**Clinical trial registration:**

https://www.medicalresearch.org.cn/, identifier MR-31-24-014367.

## 1 Introduction

The gut microbiota is a complex microbial network that is closely related to human health. Gut microbiota significantly influences intestinal barrier function and the maturation of intestinal immunity, thus protecting against pathogen invasion and colonization (Caballero-Flores et al., 2023). In addition, the gut microbiota performs a major role in the absorption and metabolism of nutrients, hormone secretion, and toxin degradation (Li et al., 2022b). Altered gut microbial balance can lead to diseases like intestinal inflammation, kidney disease, autoimmune disorders, and a higher risk of Clostridium difficile infection (Carding et al., 2015; Becattini et al., 2016; Aliberti et al., 2019; Ling et al., 2022). Antibiotics are the main strategies for fighting against infectious diseases, but they can affect the commensal microbiota (Ramirez et al., 2020). The intestine is the most concentrated place for bacteria in the body and is often affected by antibiotics (Dinan and Dinan, 2022). Many studies have confirmed that antibacterial agents can disrupt the human gut microbiota. A broad-spectrum 4-day antibiotics course with vancomycin, gentamycin and meropenem induced shifts in normal human gut microbiota composition (Mikkelsen et al., 2015). In healthy volunteers, a 5–10day course of ciprofloxacin or clindamycin resulted in a drop in microbial diversity and shifts in community composition (Haak et al., 2019). Ceftazidime/avibactam had a significant ecological impact on the normal human gut microbiota (Rashid et al., 2015). In critically ill patients, anti-anaerobic antibiotics are particularly disruptive to gut microbiota, leading to an increased risk of hospital-acquired pneumonia and nosocomial infectious diseases (Chanderraj et al., 2023). Frequent use of antibiotics in patients with chronic kidney disease (CKD) can modify the relative count of bacterial community members and reduce their diversity (Rysz et al., 2021). Gut microbiota dysbiosis and decreased renal clearance cause increased levels of uremic toxins (e.g., Trimethylamine-N-Oxide, p-cresyl and indoxyl sulfates) in CKD (Guo et al., 2024a; Xie et al., 2024a; Zhou et al., 2024a). Gut-microbiota-derived uremic toxins induce gastrointestinal inflammation, and ultimately diarrhea (Li et al., 2021; Mosterd et al., 2021; Zhou et al., 2024b). Antibiotics dramatically reduce gut microbe abundance and diversity, leading to an increased risk of adverse clinical outcomes and the development of bacterial antibiotic resistance (Palleja et al., 2018; Rysz et al., 2021; Duan et al., 2022). Therefore, studying the effects of interventions on the gut microbiota will facilitate the adoption of appropriate therapeutic regimens and reduce the aforementioned risks (Dethlefsen and Relman, 2011).

Several studies have shown that Chinese medicine intervention induced structural changes in gut microbiota (Wu and Tan, 2019; Li et al., 2024b; Xie et al., 2024b). Tanreqing injection (National Drug Permit No. Z20030054) is a commonly used drug for treating respiratory infections in China (Fan et al., 2023; Ma et al., 2024). It has several activities such as antibacterial, antiviral and anti-inflammatory (Li et al., 2024a; Guo et al., 2024b). Since Tanreqing injection (TRQ) first became commercially available in 2003, it has been recommended by many clinical guidelines and treatment protocols, such as Guideline on treating community-acquired pneumonia and the COVID-19 Diagnosis and Treatment Guidelines of China (Chun et al., 2021; Wang et al., 2023). TRQ is a standardized formulation consisting of *Radix scutellariae, Flos lonicerae, Fructus forsythiae, Fel ursi, and Cornu gorais*, with *baicalin, chlorogenic acid*, *ursodeoxycholic acid*, and *chenodeoxycholic acid* as the major bioactive constituents (Wang et al., 2021; Xie et al., 2021). TRQ has good antibacterial activity, it not only affects the expression of virulence genes in *Staphylococcus aureus* but also targets cell division to inhibit cell growth, ultimately leading to cell death (Yang et al., 2022). Meanwhile, TRQ can also inhibit the quorum sensing system of *Pseudomonas aeruginosa*, the formation of *Klebsiella pneumoniae* biofilm, and the drug resistance of *Pseudomonas aeruginosa* (Li et al., 2024a; Yang et al., 2020; Zhang et al., 2024). TRQ is an effective drug for combating bacterial infections, its standard treatment involves a 7-day course of 20 ml intravenous doses for adults. Recently, some studies have found that compared to antibiotics alone, TRQ combined with antibiotics has higher antibacterial activity against respiratory infections, particularly against drug-resistant strains such as Streptococcus pneumoniae and Staphylococcus aureus (Pan et al., 2016; Zhang et al., 2019; Li et al., 2022a). The combination treatment has become a popular option due to improved efficacy and reduced drug resistance (Tong et al., 2021; Zhou et al., 2021; Peng et al., 2022; Han et al., 2023). Although TRQ is a commonly used drug for the treatment of respiratory infections, its effect on gut microbiota is unknown.

The study aimed to assess the effect of TRQ on the gut microbiota of healthy adult volunteers at the recommended dose and regimen, which will facilitate the safe use of TRQ clinically.

## 2 Methods

### 2.1 Study design

Inclusion criteria for men and women were age 18 to 45 years and non-clinically significant findings in the medical history, physical examination, vital signs, electrocardiogram and blood laboratory results. Each volunteer had a body mass index (BMI) between 19 and 26kg/m^2^ (**Table 1**). The volunteers were willing to take adequate contraceptive measures during the entire study period and for 90 days after completion of the study. Volunteers understood and signed an informed consent form at the screening visit prior to any investigational procedure. Exclusion criteria were as follows: underlying known or previous clinically significant oncologic, pulmonary, hepatic, gastrointestinal, cardiovascular, hematologic, metabolic, neurological, immunologic, nephrological, endocrine, or psychiatric disease or current infection; drug or food allergy, drug or alcohol abuse; persons who had used drugs, vitamins, dietary supplements or herbal supplements within 14 days before the first dose of study drug; consumption of Seville oranges or products containing Seville orange components, grapefruit, grapefruit juice, or juices containing grapefruit, within 14 days before the first dose of the study drug; pregnant, risk of becoming pregnant, or breastfeeding during the study period; persons with a positive test for human immunodeficiency virus, hepatitis B virus surface antigen, or anti-hepatitis C virus antibodies. They were secondary excluded if they did not provide any fecal sample before the first dose of the study drug, or if more than 1 fecal sample was missing between day 1 and day 7 after treatment initiation.

**Table 1:** Age and BMI statistics of healthy volunteers.

Target	Average	Male	Female
AGE	28.36 ± 4.04	27.83 ± 3.09	29 ± 4.43
BMI	22.18 ± 1.84	21.88 ± 2.09	22.54 ± 1.03

### 2.2 Ethics and treatments

**Figure 1:**
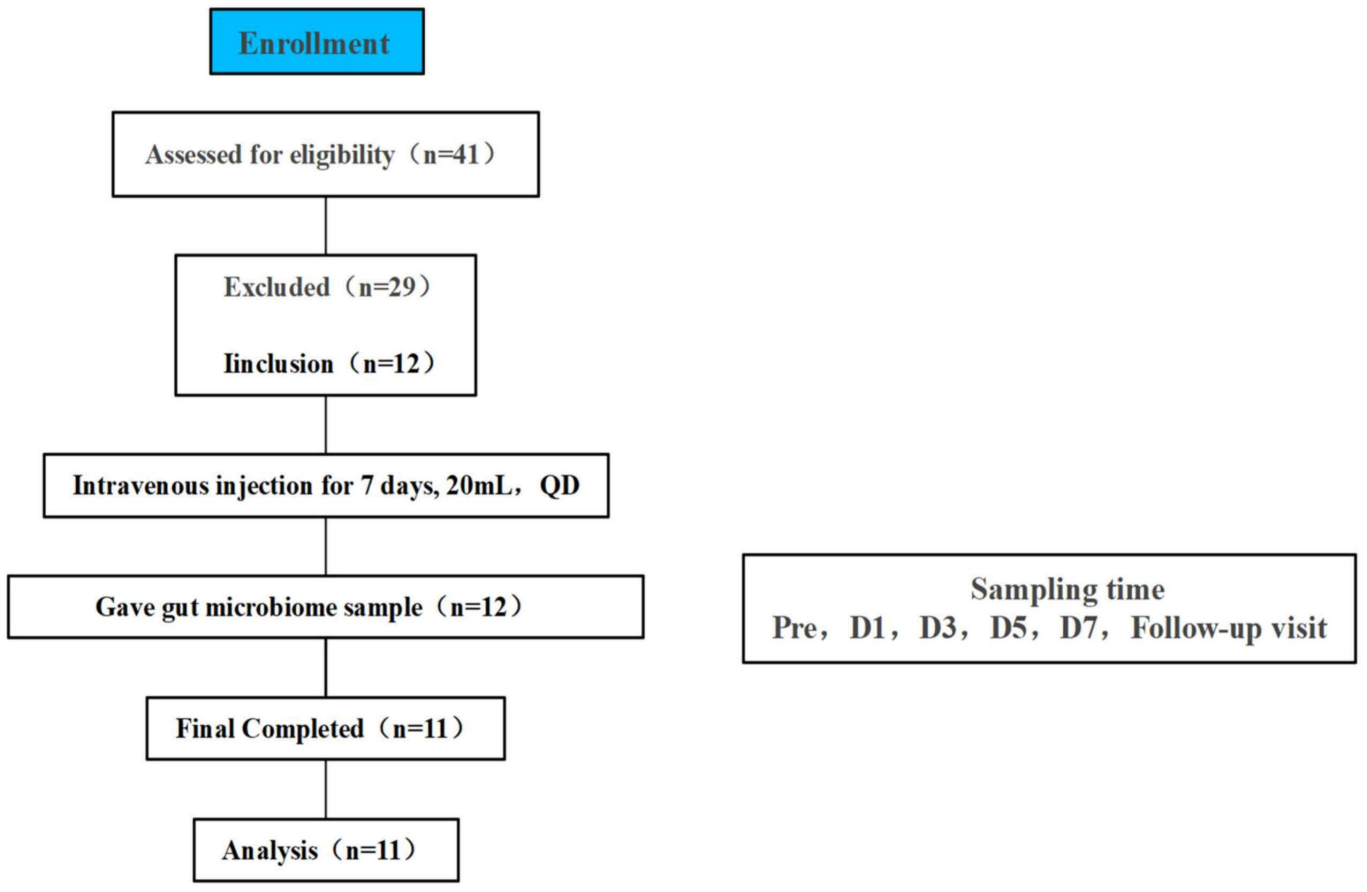
Flowchart of study subjects, a total of 41 volunteers were recruited for initial screening, 12 healthy volunteers participated in this study through the screening conditions, 1 volunteer did not collect a stool sample at the time of Pre therefore 11 people were finally included in the final analysis, TRQ was administered intravenously for 7 consecutive days at 20 mL once daily.

We recruited 12 adult healthy volunteers (6 males, 6 females) for an open, single-center clinical trial at the Clinical Research Center of Shuguang Hospital, Shanghai University of Traditional Chinese Medicine, Shanghai, China from August to September 2023 (**Figure 1**). All volunteers signed an informed consent form before screening. The trial was approved by the Ethics Committee of Shuguang Hospital of Shanghai University of Traditional Chinese Medicine on June 7, 2023 (2023-1332-99-01). This trial has been registered in the national health Security information platform and medical research registration information system (no.: MR-31-24-014367). Subjects did not consume any supplements or foods during the trial that might affect gut function or microbiota. Drinking and other conditions such as uniform meals were therefore restricted for all subjects.

Volunteers were intervened by TRQ (20mL/24hours) from day1 to day7. Briefly, 20mL TRQ was mixed with 230mL 0.9% sodium chloride solution as 120 minutes intravenous infusions using an automatic high precision infusion pump. Day 1 was defined as the first day of antibiotic treatment.

TRQ (no.: 2304105) was obtained by the Shanghai Kai Bao Pharmaceutical Co. Ltd. (Shanghai, China). 0.9% Sodium chloride injection (no.: WA2306027) came from Huaren Pharmaceutical Co. Ltd(Qingdao, China)was used for dilutions.

### 2.3 Sample collection

Fecal samples were collected from healthy volunteers at six time points: before dosing (Pre), the first day of dosing (D1), third day of dosing (D3), fifth day of dosing (D5), seventh day of dosing (D7), and two days of discontinuation (Follow-up visit). Stool samples were placed in sterile containers and transferred to a -80°C refrigerator within 30 minutes of defecation for storage until processing.

### 2.4 Sample processing and sequencing

Total microbial genomic DNA was extracted from the above samples using the OMEGA Stool DNA Kit (Omega Bio-tek, Georgia, U.S.) according to the manufacturer’s instructions. The quality and concentration of DNA were determined by 1.0% agarose gel electrophoresis and a NanoDrop^®^ ND-2000 spectrophotometer (Thermo Scientific Inc., USA) and kept at -80 °C prior to further use. The hypervariable region V3-V4 of the bacterial 16S rRNA gene sequencing were amplified with primer pairs 338F (5’-ACTCCTACGGGAGGCAGCAG-3’) and 806R(5’-GGACTACHVGGGTWTCTAAT-3’) by an ABI GeneAmp^®^ 9700 PCR thermocycler (ABI, CA, USA) (Liu et al., 2016). The PCR mixture consisted of 4 μL 5× Fast Pfu buffer, 2 μL 2.5 mM dNTPs, 0.8 μL each primer, 0.4 μL FastPfu polymerase, 0.2 μL BSA, 10 ng template DNA, and ddH2O up to 20 µL. PCR cycling conditions: initial denaturation at 95°C for 3 min; 27 cycles of 95°C for 30 s, 55°C for 30 s, and 72°C for 45 s; final extension at 72°C for 10 min, and hold at 10°C. All samples were amplified in triplicate. The PCR product was extracted from 2% agarose gel and purified. Then quantified using Quantus™ Fluorometer (Promega, USA).

Purified amplicons were pooled in equimolar amounts and paired-end sequenced on an Illumina PE300 platform (Illumina, San Diego, USA) according to the standard protocols by Majorbio Bio-Pharm Technology Co. Ltd. (Shanghai, China).

### 2.5 Data processing and statistical analysis

Bioinformatic analysis of the gut microbiota was carried out using the Majorbio Cloud platform (https://cloud.majorbio.com). Based on the OTUs information, rarefaction curves and alpha diversity indices including observed OTUs, Shannon index and Simpson index and Chao1 index were calculated with Mothur v1.30.2 (Schloss et al., 2009). Sample microbial community similarities were analyzed with PCoA using weighted and unweighted Unifrac distances, via the Vegan v2.4.3 package. Treatment variation and significance were assessed with the PERMANOVA test using the Vegan v2.4.3 package. Partial least square discriminant analysis (PLS-DA) was used to analyze small between-group differences and large within-group variations, effectively identifying the affecting variables. PcoA and PLS-DA were utilized to quantify compositional differences, thus indicating the beta diversity within the bacterial community samples.

The linear discriminant analysis (LDA) effect size (LEfSe) was used to analyze the dominant flora to detect intergroup differences in the sample microbiota. LEfSe analysis (http://huttenhower.sph.harvard.edu/LEfSe) identified significantly abundant bacterial taxa from phylum to genera among groups (Segata et al., 2011); PICRUSt2 analysis predicted the functional composition of microbial genomes by calculating Clusters of Orthologous Groups (COG) and Kyoto Encyclopedia of Genes and Genomes (KEGG) abundances, normalizing OTU tables, and comparing green gene ID. Bugbase phenotype prediction utilized pre-calculated files to predict microbial phenotypes post-OTU normalization. To control for the risk of false positives due to multiple testing, False Discovery Rate (FDR) correction was applied in this study. All p-values from statistical tests were adjusted using FDR. In this paper, only those *P* < 0.05 were considered statistically significant.

## 3 Result

Eleven subjects were included in the analysis, and one of them was not sampled at Pre due to the lack of pre-and post-test mechanical controls. No adverse reactions were observed at any time point during the study.

### 3.1 α- and β- Diversities

**Figure 2:**
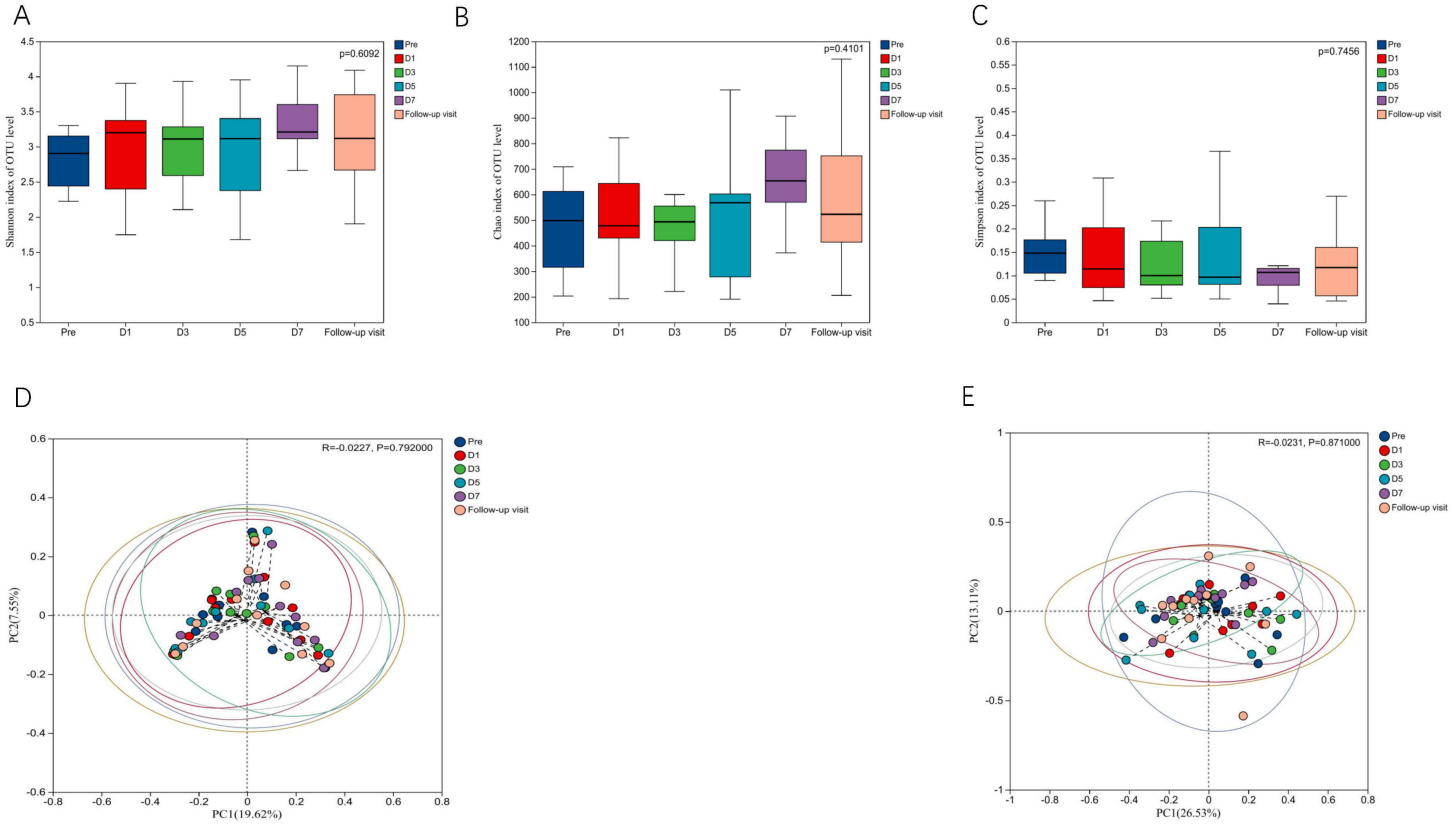
Effects of TRQ on α and β diversity of gut microbiota in healthy volunteers. **(A)** Shannon index and **(B)** Simpson index and **(C)** Chao1 index were used to assess species richness and evenness to indicate changes in α diversity levels, and data are shown in a box plot with the middle horizontal line representing the median. **(D)** Unweighted and **(E)** weighted PCoA were used to show the similarities and differences between samples at each time point, and to evaluate the changes in gut microbial community structure by looking at the clustering and dispersion of sample points. Changes in gut microbial community structure were evaluated by testing the clustering and dispersion of sample points, and the samples at different time points were represented by different colors.

**Figure 3:**
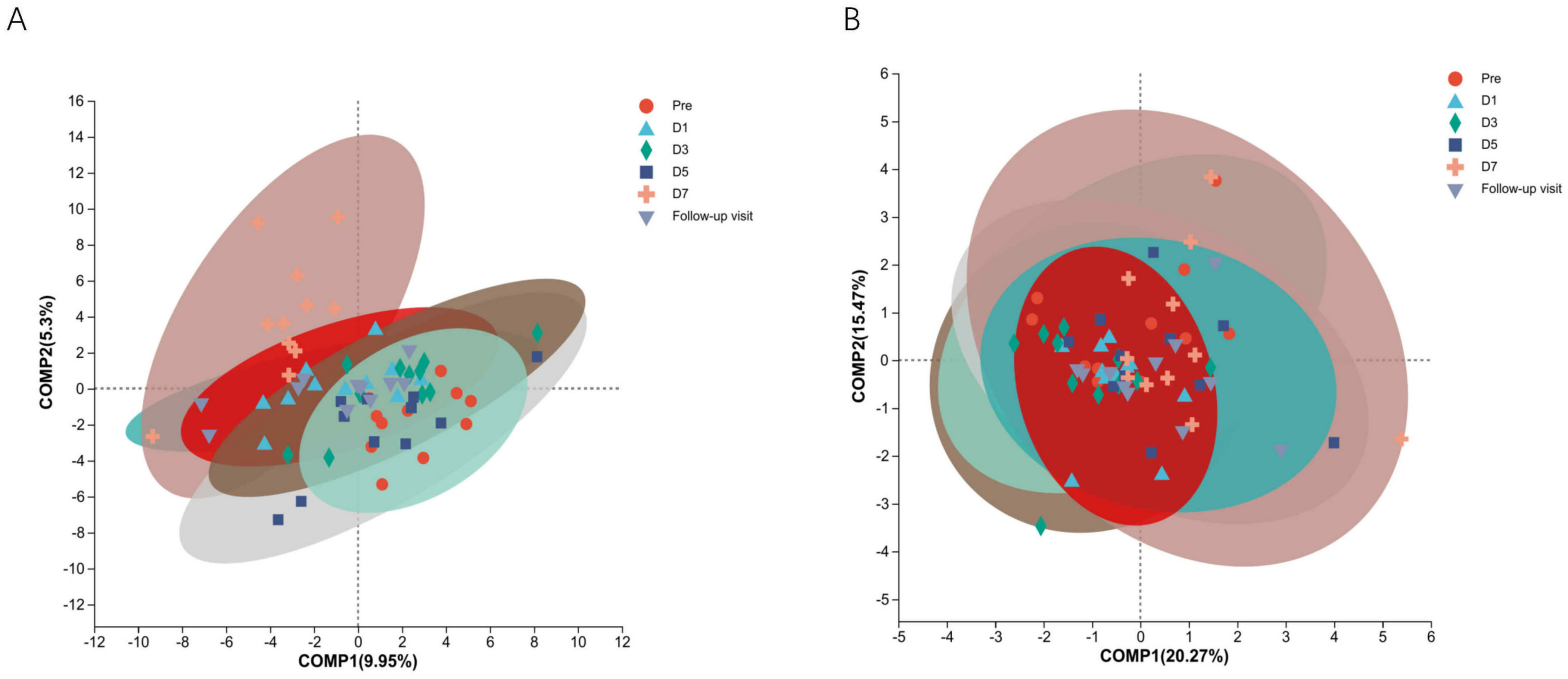
The study utilized Partial Least Squares Discriminant Analysis (PLS-DA) to identify the structural differences in microbial communities between groups, specifically samples from different time points. **(A)** Shows information on structural differences between time point samples at the genus level; **(B)** Shows information on structural differences between time point samples at the phylum level. Different colors and shapes represent different time points and samples.

To know the effect of TRQ on gut microbial community structure, we performed 16S rRNA gene sequencing and analysis. Using the α-diversity index to assess species richness and evenness (Shannon and Simpson and Chao1 diversity indices). Species diversity during administration and two days after drug withdrawal with median Shannon index values of 2.90, 3.20, 3.10, 3.11, 3.20 and 3.12. These changes were not statistically significant (*P*> 0.05) (**Figures 2A–C**). Furthermore, TRQ had no significant effect on β-diversity of microbial communities (**Figures 2D, E**). Sample dispersion across time points was not significant in both weighted and unweighted Unifrac distances analyses. Besides, although the indexes of richness and diversity failed to show a notable change, partial least squares-discriminant analysis (PLS-DA) plots showed that a distinct dissociation trend emerged on D7 at the genus level (**Figure 3A**), returning to baseline two days after discontinuation. However, no significant sample dispersion at the phylum level (**Figure 3B**). For the genus level, subjects L05, L08 and L09 exhibited pronounced dissociation on D7. But the PERMANOVA test analysis did not reveal any significant differences between the groups. Similarly, the LEfSe tool did not identify any distinctive microbial biomarkers (**Supplementary Figure S1**, LDA > 2).

### 3.2 Effects on microbiota community composition

**Figure 4:**
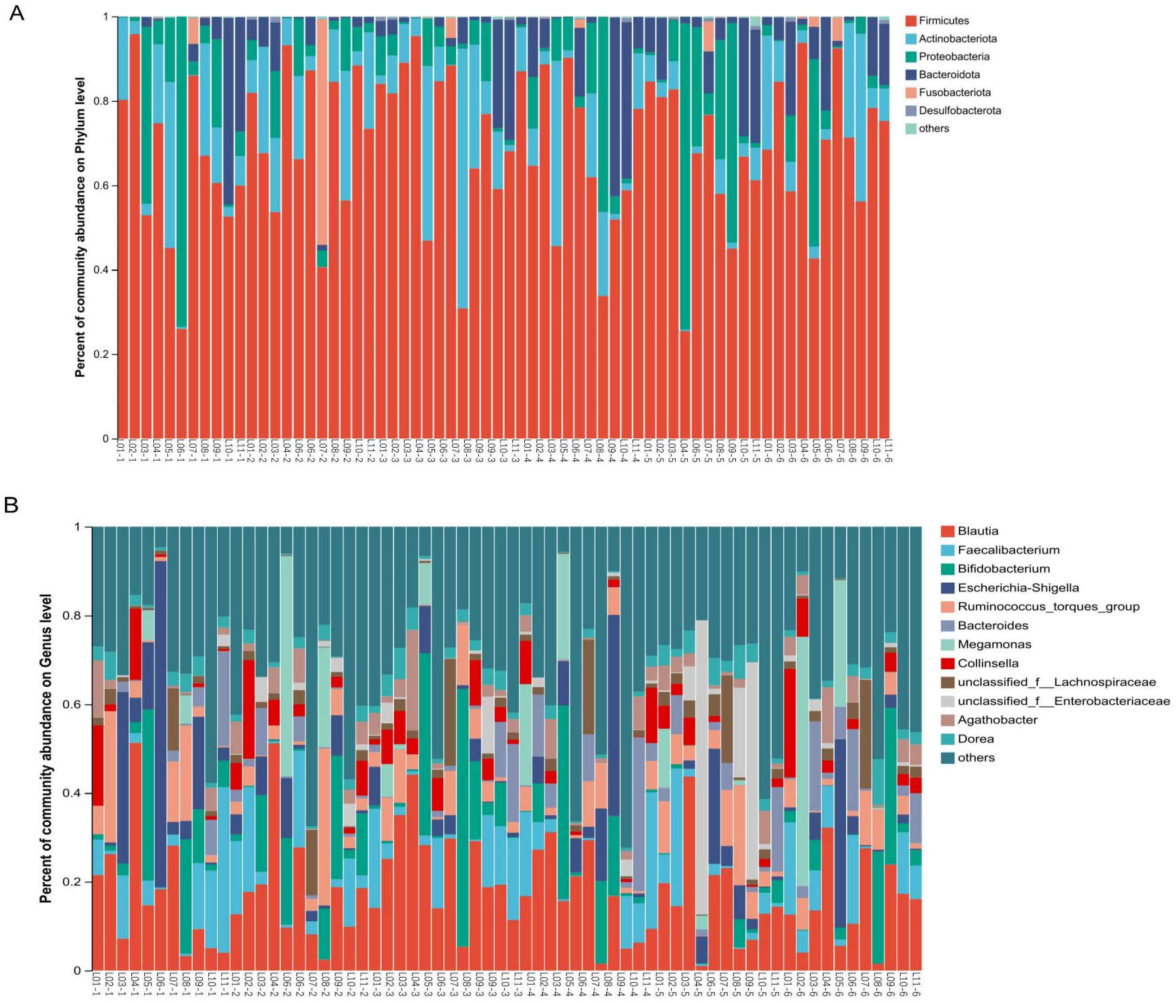
Effects of TRQ use for one week on gut microbial composition (phylum and genus level) in healthy subjects. Fecal samples were collected at various time points: before the trial began (Pre), on the first day of administration (D1), on the third day of administration (D3), on the fifth day of administration (D5), on the seventh day of administration (D7), and two days after discontinuation of the drug (Follow-up visit). Each bar represents a single sample, and the names of the upper and lower samples correspond to each other. The colors used to distinguish Phylum **(A)** and Genus **(B)** represents the occupancy. The top 6 bacterial phyla in abundance were at the Phylum level, while the top 12 bacterial genera in abundance were at the Genus level (the others were combined with a relative abundance of less than 1%). The L represents the subject and the number represents the sampling time (1-Pre, 2-D1, 3-D3, 4-D5, 5-D7, 6-Follow up visit).

β-diversity analysis indicates changes in gut microbial community structure on D7. To explore the potential reasons for these changes, we examined the major gut microbial flora across all taxonomic levels. The dominant flora has an important effect on the stability of microbial communities. Our study found that the gut microbiome mainly consists of four phyla: Firmicutes, Actinobacteriota, Proteobacteria and Bacteroidota (**Figure 4A**). In exploratory analysis of mean abundance at the phylum level, it was observed that Firmicutes increased on D1, D3, and the follow-up visit, while Actinobacteriota decreased on D7. Additionally, there was a decrease in Proteobacteria during dosing (**Table 2**). Class, order, and family level analyses showed increases in Clostridia, Lachnospirales, and Lachnospiraceae on D3. Gammaproteobacteria, Enterobacteriales and Enterobacteriaceae decreased during administration; Actinobacteria, Bifidobacteriales and Bifidobacteriaceae were reduced on D7. Two days after drug withdrawal, there was an increase in Negativicutes, Veillonellales-Selenomonadales and Selenomonadaceae (**Supplementary Figures S2-S4**, **Supplementary Tables S1-S3**). At the genus level, *Bifidobacterium* decreased by 6.887% on D7, specifically in subjects L05 and L08. *Escherichia-Shigella* decreased by 10.487%, especially in subjects L03, L06 and L09. Unclassified_f_Enterobacteriacea increased by 13.0611%, particularly in subjects L05, L08 and L09 (**Figure 4B**, **Supplementary Table S4**). No differences in the aforementioned species were observed after multiple tests analysis.

**Table 2:** Mean relative abundance (%) and *p* of the phylum.

Phylum	Pre	D1	D3	D5	D7	Follow-up visit	*P*
Firmicutes	63.70	72.09	72.81	66.27	66.09	72.01	0.791
Actinobacteriota	12.09	13.57	16.94	10.19	4.75	11.95	0.676
Proteobacteria	15.66	5.78	5.19	9.42	18.27	6.58	0.791
Bacteroidota	7.77	3.36	4.25	13.52	9.64	8.33	0.676
Fusobacteriota	0.59	4.87	0.46	0.20	0.67	0.75	0.884
Desulfobacterota	0.05	0.19	0.17	0.22	0.25	0.22	0.694

Significant variations in bacteria at the phylum to genus levels were observed, including Leuconostocaceae, Bacillaceae, *Weissella*, and Cyanobacteria, accounting for 0-0.3% of the total abundance (*P* < 0.05, **Supplementary Table S5**). To minimize mutual interference among samples at different time points, two-group difference tests were conducted separately on species composition for Pre, D7 and Follow-up visit. The results from multiple tests analysis showed no differential species detected.

**Figure 5:**
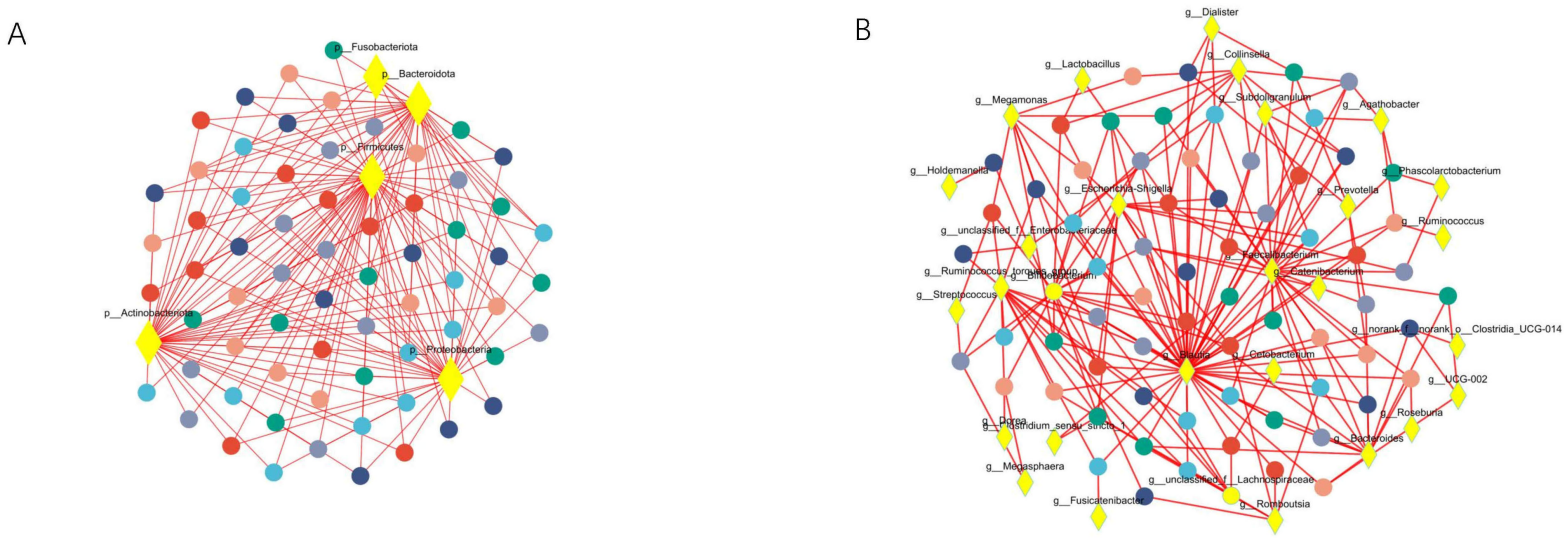
Co-occurrence network analysis was performed to observe the relationship between species and samples, so as to identify the central species in the community. **(A)** Represents the results of the analysis at the phylum level. **(B)** Represents the results at the genus level. The diamond-shaped nodes in yellow represent species at the taxonomic level. Circular nodes of different colors represent samples at different time points, and edges represent correlations.

Co-occurrence network analysis was conducted at both the phylum and genus levels to assess the significance of dominant gut microflora (**Figure 5**). At the phylum level, species with high weighted degrees were identified, including Firmicutes, Actinobacteriota, Proteobacteria, and Bacteroidota. Genus-level species *Blautia, Faecalibacterium, Bifidobacterium, Escherichia-Shigella, Ruminococcus_torques_group, Bacteroides, Megamonas, Collinsella*, unclassified_f_Lachnospiraceae, and unclassified_f_Enterobacteriaceae exhibited high weighted-degree (**Table 3**). As illustrated in **Figure 4**, these species have a vital role in the community and have a significant impact on its structure and function.

**Table 3:** Weighted-degree of species.

Node-name	Weighted degree
Firmicutes	1857057
Actinobacteriota	312447
Proteobacteria	273836
Bacteroidota	210764
*Blautia*	477152
*Faecalibacterium*	207186
*Bifidobacterium*	206035
*Escherichia-Shigella*	180065
*Ruminococcus_torques_group*	151624
*Bacteroides*	112961
*Megamonas*	103416
*Collinsella*	93778
unclassified_f_Lachnospiraceae	85894
unclassified_f_Enterobacteriaceae	83433

### 3.3 Prediction of changes in the function of the intestinal microbiota

This study used the PICRUSt2 tool to predict changes in gut microbiota function before and after TRQ administration. KEGG pathway analysis results showed the abundance of predicted functions related to metabolism, global and overview maps, carbohydrate metabolism, ko01100, and ko01110 were high (**Supplementary Figures S5-S7**). COG analysis revealed high abundance in amino acid transport and metabolism, carbohydrate transport and metabolism, translation, ribosomal structure and biogenesis, as well as transcription demonstrated (**Supplementary Figure S8**). No statistically significant differences in functional categories were detected before and after TRQ treatment, according to the Kruskal-Wallis H test. BugBase phenotype prediction indicated a higher predominance of Anaerobic and Gram-Positive, with no statistical significance found for all assessed phenotypic categories (**Supplementary Table S6**).

## 4 Discussion and conclusion

Gut microbiota performs several vital functions by aiding digestion, stimulating and regulating the immune system and preventing the growth of pathogens (Scott et al., 2024). Therapeutic and prophylactic drugs with antibacterial properties can disrupt the normal gut microbiota structure that have both short- and long-term health consequences (Rashid et al., 2016; Ramirez et al., 2020). One study documented healthy volunteers treated for 1 week or less with antibiotics reported effects on their bacterial flora that persisted 6 months to 2 years after treatment, including a dramatic loss in diversity as well as in representation of specific taxa, and insurgence of antibiotic-resistant strains (Becattini et al., 2016). Gut microbiota dysbiosis also increases the generation of uremic toxins, reduces the production of short-chain fatty acids, and affects metabolism such as adenine, which may increase an individual’s risk of metabolic syndrome, renal injury, and inflammatory bowel diseases, as well as susceptibility to infectious diseases (Chen, 2020; Ramirez et al., 2020; Rysz et al., 2021; Li et al., 2023; Zhou et al., 2024a). Knowledge of how these drugs interact with the normal gut microbiota can help clinicians select appropriate drugs (Feng et al., 2020). TRQ is a drug with antibacterial activity that is widely used in the treatment of respiratory infections, and understanding its potential effects on the gut microbiota is necessary.

In this study, we observed that the use of TRQ did not result in significant changes in the gut microbiota of healthy individuals. Antibiotic treatment reduces the diversity of gut microbiota species, and a loss of species diversity is a common feature of a disturbed gut microbiota (Lange et al., 2016; Ramirez et al., 2020). Alpha-diversity is used to evaluate the richness of each sample, and its decrease is associated with disease (Li et al., 2022b). It has been reported that within a few days after treatment with antibiotics such as Cefprozil, ciprofloxacin, vancomycin, and metronidazole, the Shannon and Chao1 indices of healthy individuals significantly decrease, indicating that antibiotics can markedly affect the species diversity of the gut microbial communities. Additionally, significant differences were observed between samples before and after antibiotic treatment in both weighted and unweighted PCoA, revealing that the beta diversity of the microbial communities was affected by the antibiotics (Burdet et al., 2019; Haak et al., 2019). Our study showed that in alpha-diversity analysis, Shannon, Chao1, and Simpson indices did not find any significant differences, and no significant dispersion occurred in the weighted and unweighted PCoA (*P*>0.05). These results indicates that no bacterial species were extinguished by TRQ.

PLS-DA can offer a discerning visual analysis, more intuitively find differences between groups (Zhuang et al., 2016). Genus-level analysis revealed a separation trend in the PLS-DA model, returning to baseline after discontinuation. This implies potential alterations in the gut microbial community structure at a more detailed classification level. Significantly discrete sample points in the model corresponded to subjects L05, L08, and L09. The fluctuations in the abundance of unclassified_f_Enterobacteriaceae, *Bifidobacterium, and Escherichia-Shigella* were observed in these three subjects only. These changes indicate individual differences. The segregation trend in the PLS-DA model likely stems from the discrete sample points of subjects L05, L08, and L09. However, no statistically significant were observed. Bacteria with significant changes at phylum and genus levels accounted for less than 1% of total abundances (**Supplementary Table S5**), suggesting a minimal impact on community structure. These findings indicate that TRQ does not significantly affect the gut microbial community’s overall structure.

In the context of the KEGG database, Pathway1, Pathway2, and Pathway3 together depict a multilayered network ranging from broad biological functions to specific metabolic mechanisms (Antonov et al., 2008). Analysis of KEGG Pathway1 of gut microbial function, which are generally divided into six major categories: metabolism, genetic information processing, environmental information processing, cellular processes, human diseases, and organismal systems (Xie et al., 2024b). At this macroscopic level, no significant difference in results was found. Consistent with Pathway1, analyses of Pathway2 and Pathway3 also indicated no significant functional alterations. Furthermore, to substantiate our findings, we utilized the COG database for functional predictions and the Bugbase platform for phenotypic assessments of gut microbes. These findings revealed no significant differences, thereby indicating the microbial community’s functional and phenotypic stability under the study’s conditions. However, due to the limitations of PICRUSt2, the analysis of predicted gut microbiota function only provided some preliminary results. Functional changes in the gut microbiota should be confirmed by combining metabolomics and macro genomics in the future.

TRQ is widely used to treat respiratory infections in China (Guo et al., 2024b). Recently, TRQ combined with antibiotics is popular in the treatment of respiratory infection due to its improved efficacy and reduced drug resistance (Peng et al., 2022; Han et al., 2023). Understanding the effects of TRQ on the gut microbiota is important for its safe application. This study first reported the effect of TRQ on the gut microbiota. Family genetic background, dietary habits and lifestyle can influence the gut microbiota and lead to inter-individual differences (Anwar et al., 2021), so we adopted a self-controlled clinical trial design. Self-controlled clinical trial design can not only effectively control individual differences and reduce sample size, but also improve the internal validity of the study to draw more reliable conclusions (Iwagami and Takeuchi, 2021). In this study we employed the most commonly used dose of TRQ, which is 20ml. The composition of gut microbiota varies dynamically, thus we selected 6 time points for fecal sample collection. The healthy, adult gut microbiome is relatively stable in an individual and resilient to perturbation (Anwar et al., 2021). The effect of TRQ on gut microbiota can be evaluated more objectively in healthy volunteers. However, our study had a relatively small sample size, and the age distribution of the participants was uneven. When applying the research results, these limitations need to be considered. Further studies will involve more participants of different age groups to better understand the effects of TRQ on the gut microbiota.

Despite these limitations, the findings of this pilot study provide valuable information for the appropriate use of TRQ. Gut microbiota dysbiosis and the rise in antibiotic-resistant strains are the major problems faced by antibiotic therapy. With the increased use of antibiotics, these problems are likely to become more acute or more prevalent in the future. Indeed, new therapeutical agents will be necessary to substitute or complement the use of antibiotic treatments. TRQ, as a natural medicine, has unique advantages in preventing and treating respiratory infections (Ma et al., 2019; Ye et al., 2020). TRQ provides an option for patients with limited treatment options due to antibiotic resistance or Gut microbiota dysbiosis.In conclusion, the study indicates that short-term use of TRQ at conventional doses may not cause perturbations to the gut microbiota in healthy adults. This finding provides some useful information for the safe use of TRQ in the treatment of respiratory infections.

## Data Availability

The raw data supporting the conclusions of this article will be made available by the authors, without undue reservation.
