# Supplementary material for: Effects of Tanreqing injection on the gut microbiota in healthy volunteers
Source: Front Cell Infect Microbiol. 2024 Oct 4;14:1428476. doi: 10.3389/fcimb.2024.1428476 (PMC11486765; doi:10.3389/fcimb.2024.1428476)
Supplement: Supplementary file 1 [file Table1.docx]

| **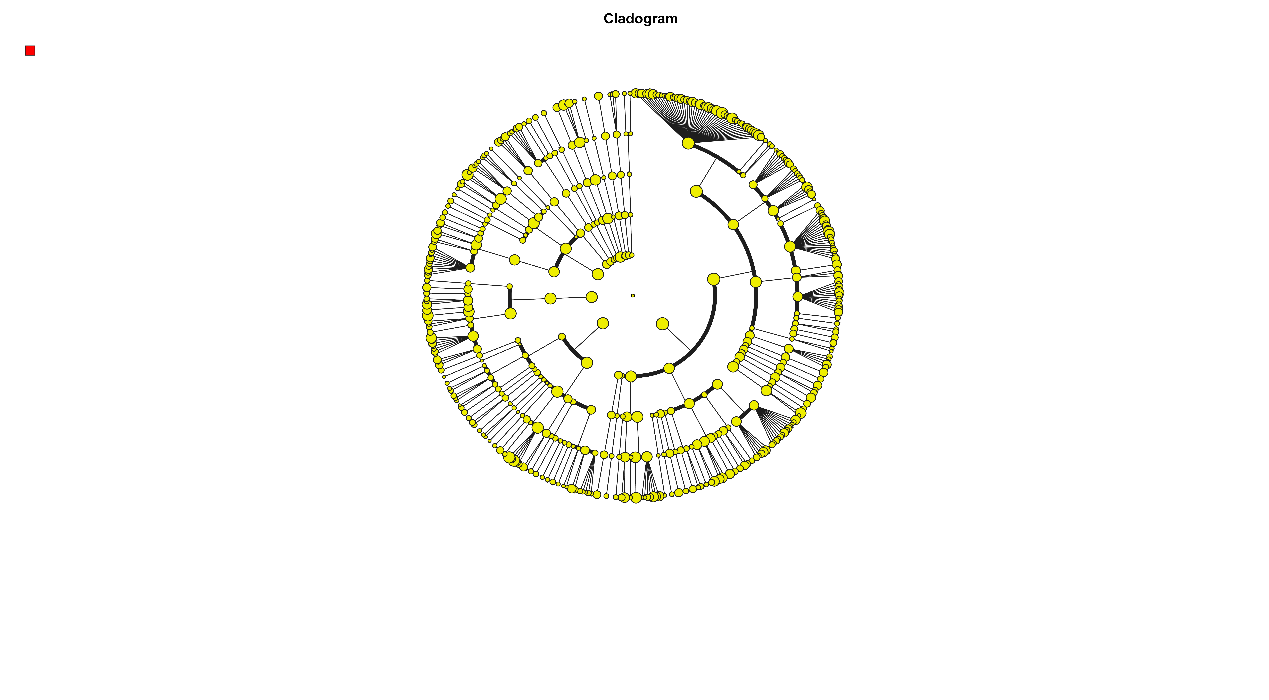** |
| --- |
| Figure S1: LEfSe cladogram representing the the level of classification from phyla to genus. The diameter of each circle is proportional to the abundance of taxa. Yellow circles indicate no significant difference, while colored nodes (shaded by green and red) indicated groups that were significantly enriched in one group compared to other groups. Branches and nodes are organized from inner circles (phylum) to outer circles (genus) according to taxonomic rank. The groups with the most significant difference in abundance were prominent in larger circles and associated with specific groups through shading. As can be seen from the diagram, there is no obvious differentiation of taxa. |


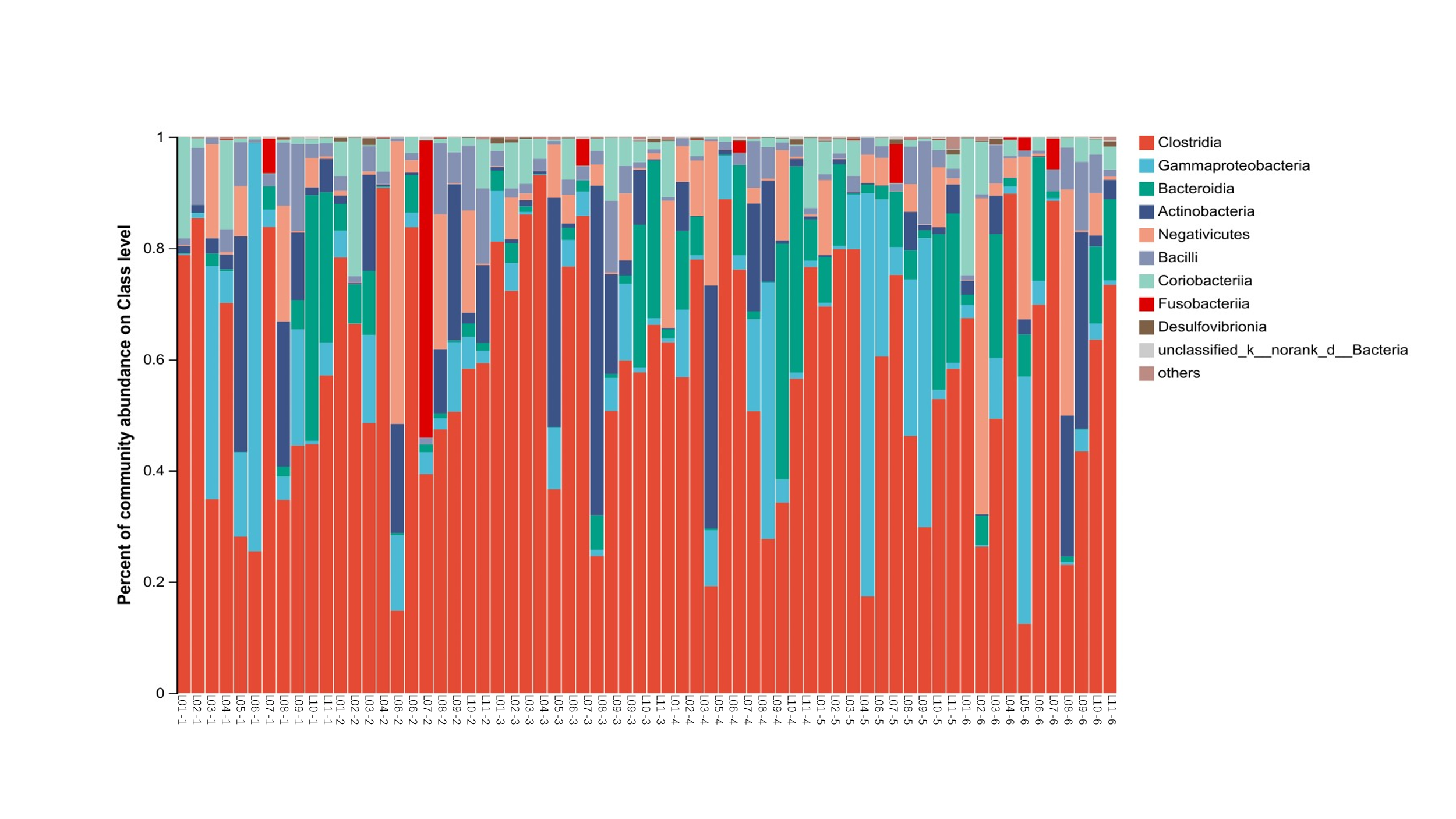


|  |
| --- |
| FigureS2：Graphical representation of the relative taxa abundance at the class level for healthy human subjects receiving TRQ. Each bar represents a single sample (single subject at each timepoint) (Mean abundance). The L represents the subject and the number represents the sampling time (1-Pre, 2-D1, 3-D3, 4-D5, 5-D7, 6-Follow up visit). |

|  |
| --- |
| 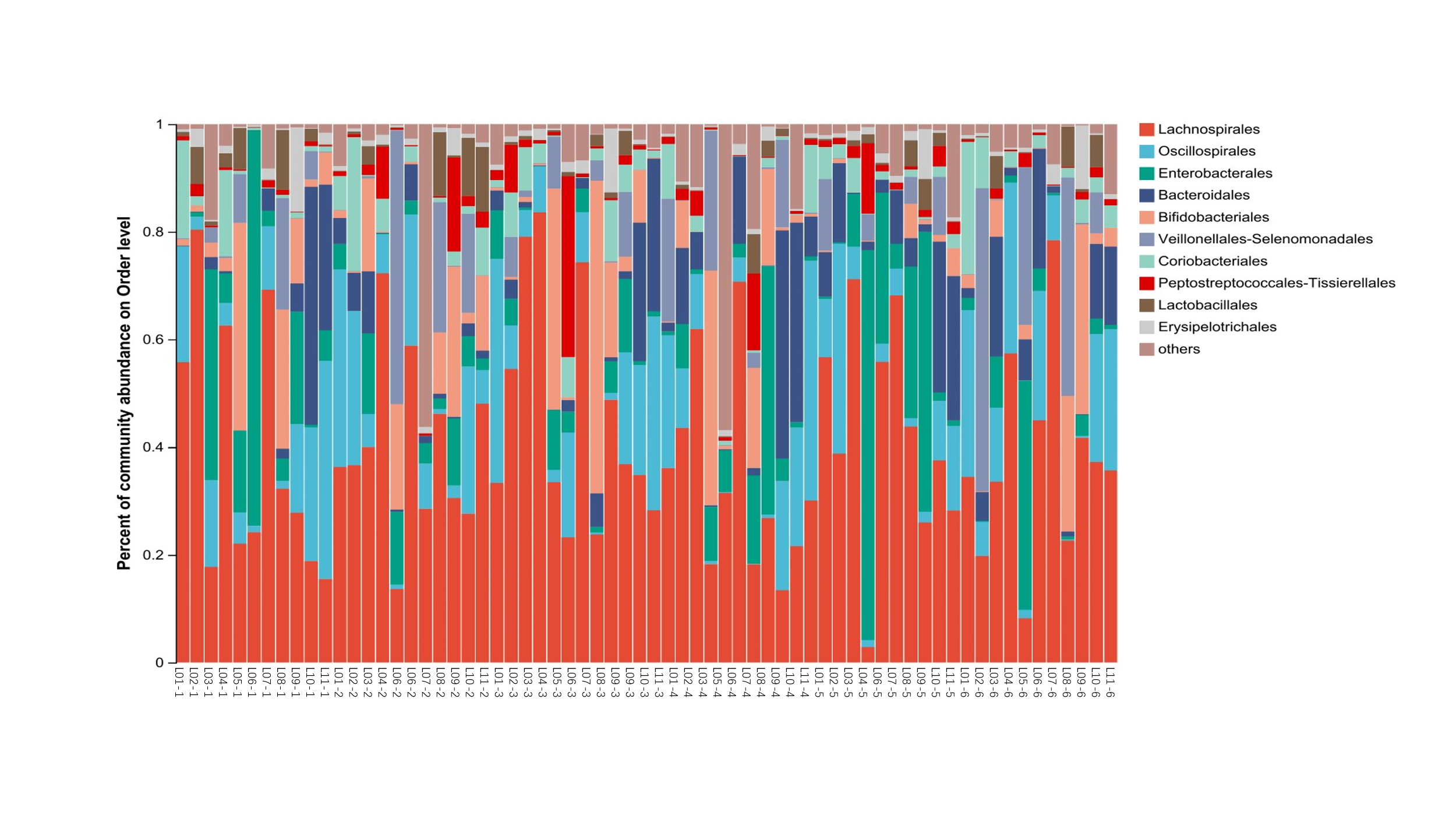  FigureS3：Graphical representation of the relative taxa abundance at the order level for healthy human subjects receiving TRQ. Each bar represents a single sample (single subject at each timepoint) (Mean abundance). The L represents the subject and the number represents the sampling time (1-Pre, 2-D1, 3-D3, 4-D5, 5-D7, 6-Follow up visit). The L represents the subject and the number represents the sampling time (1-Pre, 2-D1, 3-D3, 4-D5, 5-D7, 6-Follow up visit). |

|  |
| --- |
| 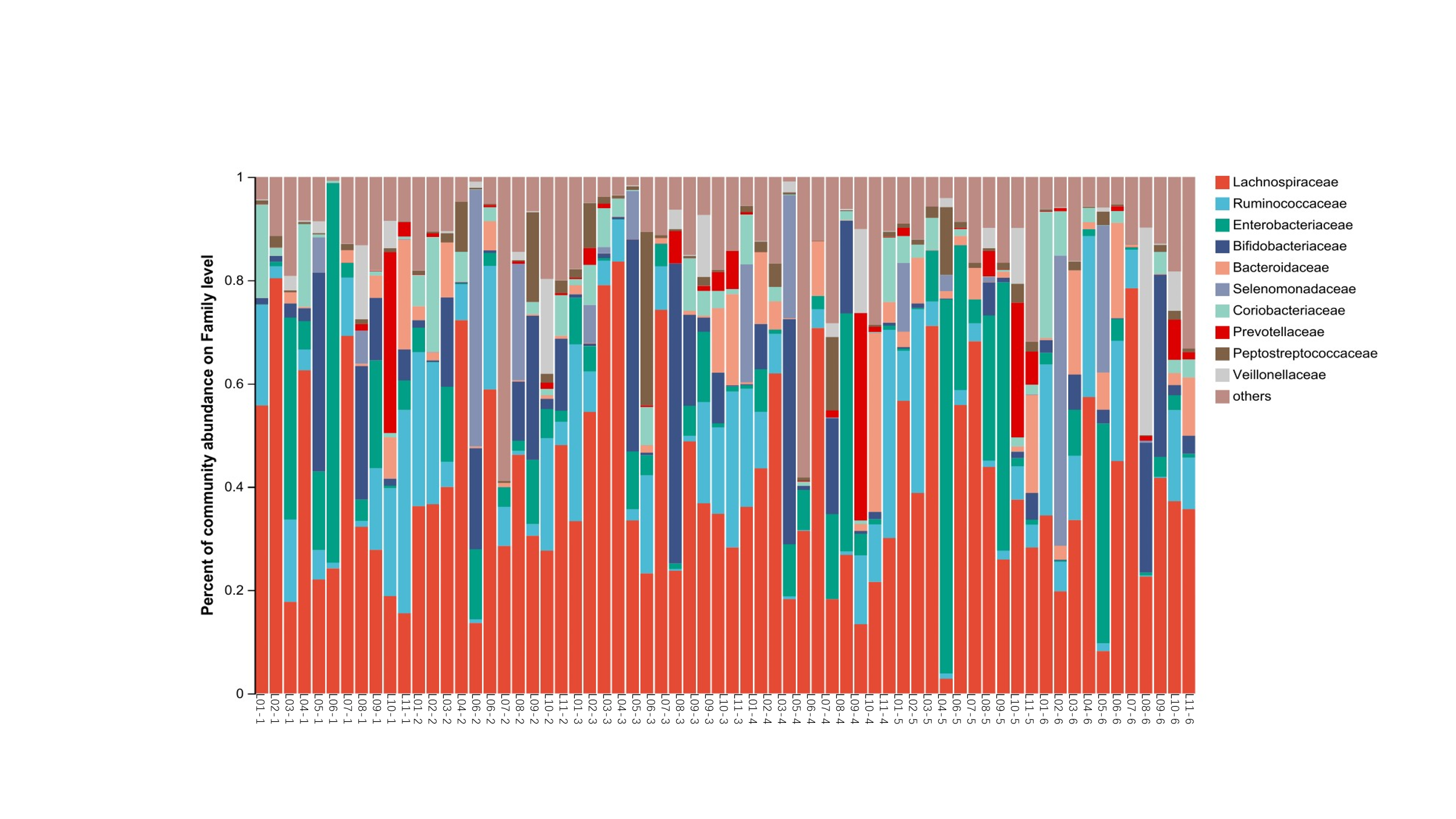  FigureS4：Graphical representation of the relative taxa abundance at the family level for healthy human subjects receiving TRQ. Each bar represents a single sample (single subject at each timepoint) (Mean abundance). The L represents the subject and the number represents the sampling time (1-Pre, 2-D1, 3-D3, 4-D5, 5-D7, 6-Follow up visit). |

| 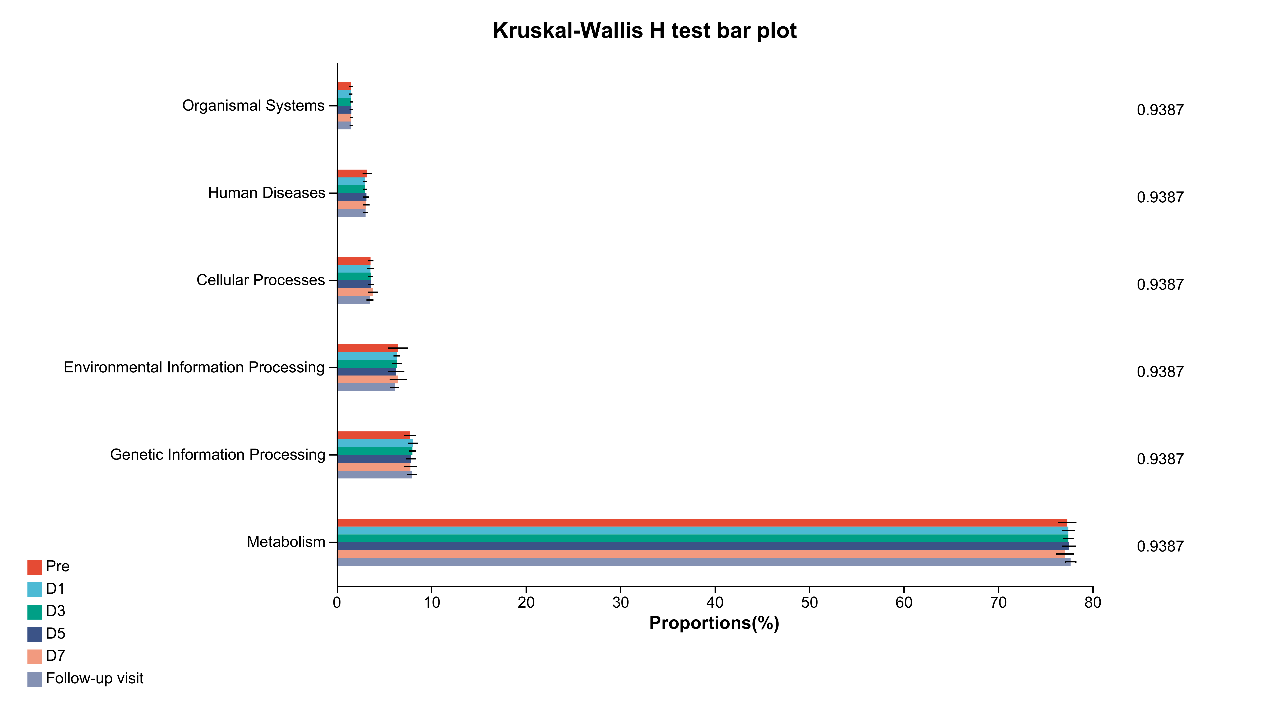 |
| --- |
| Figure S5: Comparative Analysis of Microbial Functional Pathways Over Time (pathway1). The bar plot presents the temporal dynamics of microbial functional pathways before and after TRQ treatment. Each bar represents the proportion of sequences assigned to high-level functional categories such as metabolism, genetic information processing, and organismal systems at different time points (*P*＞0.05). |

| 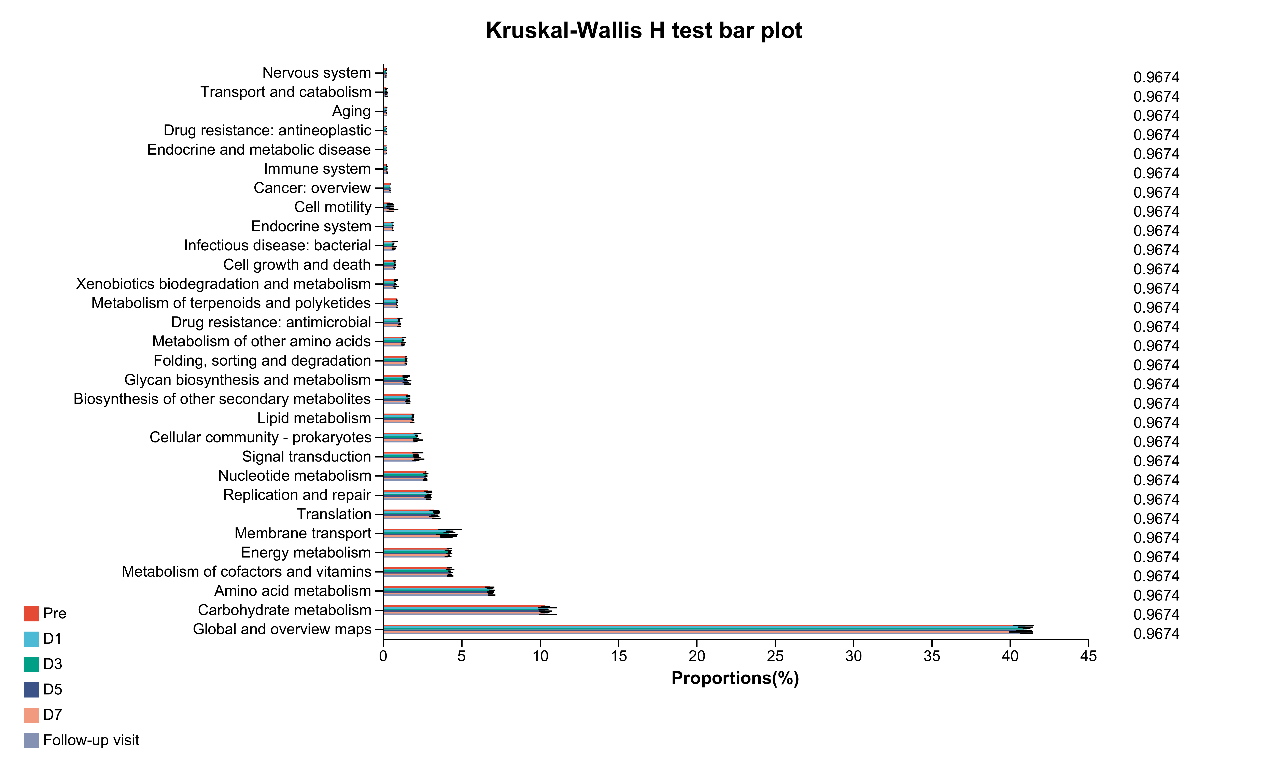 |
| --- |
| Figure S6: Comparative Analysis of Microbial Functional Pathways Over Time (pathway2). The bar chart depicts the proportional distribution of predicted microbial functional pathways at various time points before, during, and after TRQ treatment. The data indicate that there are no statistically significant shifts in the microbiome's functional capabilities as evidenced by the Kruskal-Wallis H test (*P*＞0.05). |

| 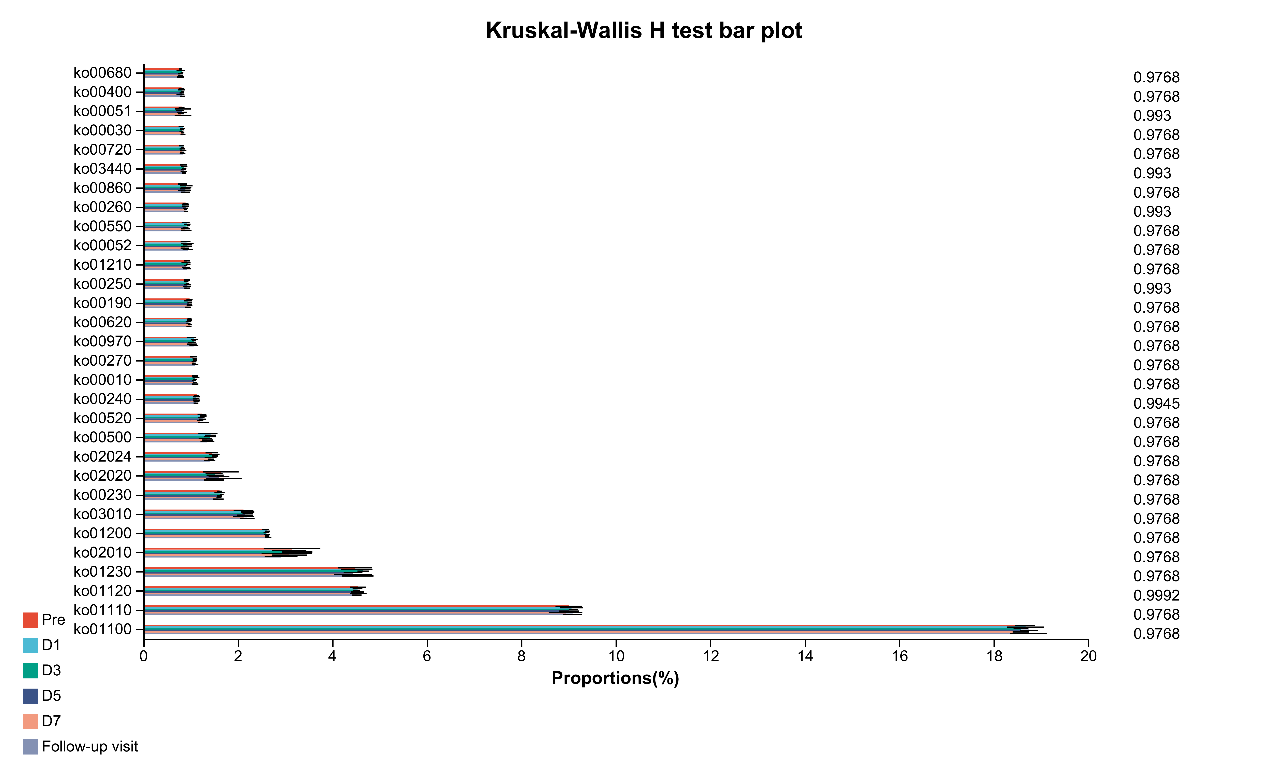 |
| --- |
| Figure S7: Comparative Analysis of Microbial Functional Pathways Over Time (pathway3). The bar graph illustrates the proportions of specific KEGG Orthology (KO) categories across various time points in relation to TRQ treatment. The stability of microbial functions is depicted, as no significant changes are indicated in the proportions of these functional categories before, during, and after treatment (*P*＞0.05). |

| 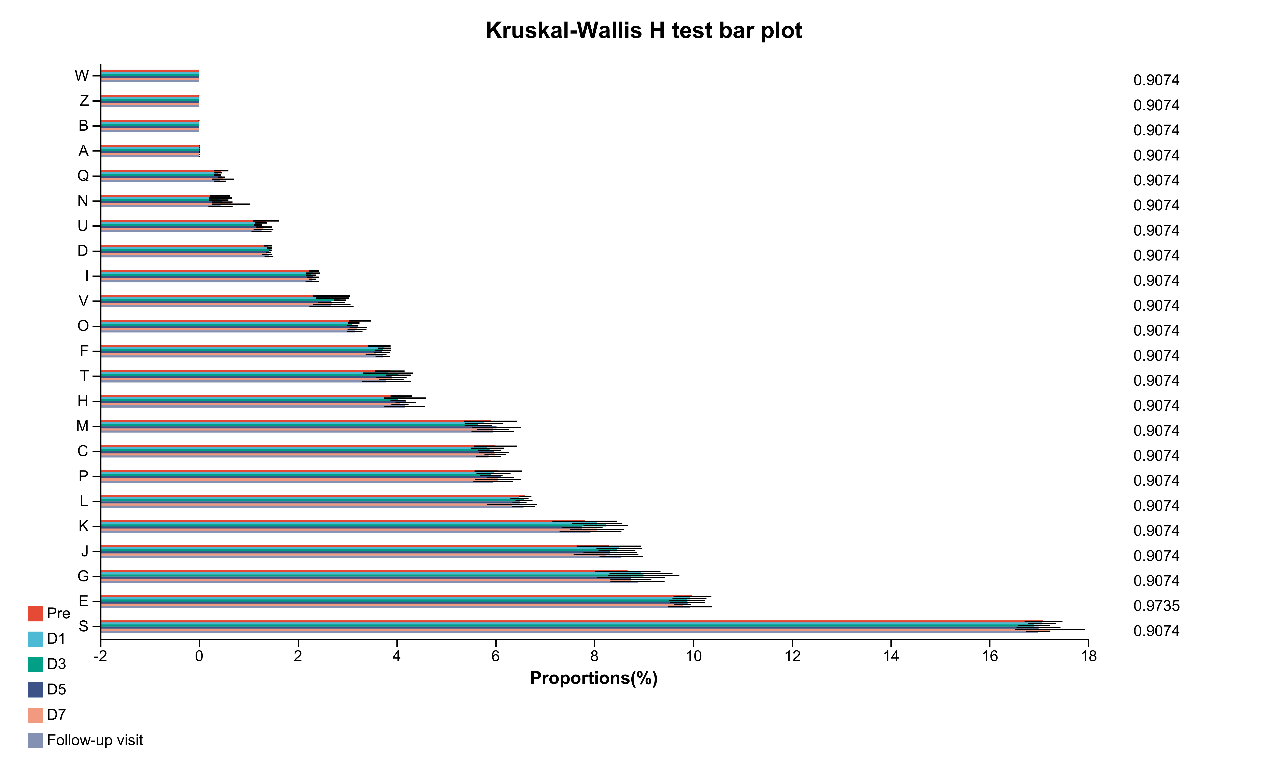 |
| --- |
| Figure S8: Distribution of Predicted COG Functional Categories Across Different Time Points. The bar graph illustrates the distribution of COG functional categories within the gut microbiome across various time points before, during (D1, D3, D5, and D7), and after TRQ treatment. Each bar represents the proportion of each COG category in the samples at different times. The differences across time points were analyzed using the Kruskal-Wallis H test (*P*＞0.05). |

TableS1：Mean relative abundance (%) and *P* of the Class

| Species name | Pre | D1 | D3 | D5 | D7 | Follow-up visit | *P* |
| --- | --- | --- | --- | --- | --- | --- | --- |
| Clostridia | 53.42 | 57.96 | 65.87 | 56.11 | 58.72 | 55.18 | 0.929 |
| Gammaproteobacteria | 15.65 | 5.77 | 5.16 | 9.40 | 18.26 | 6.57 | 0.929 |
| Bacteroidia | 7.76 | 3.36 | 4.25 | 13.52 | 9.64 | 8.33 | 0.881 |
| Actinobacteria | 8.39 | 8.65 | 12.26 | 8.50 | 1.52 | 7.16 | 0.881 |
| Negativicutes | 4.87 | 8.90 | 3.65 | 7.85 | 3.74 | 12.86 | 0.929 |
| Bacilli | 5.38 | 5.17 | 3.23 | 2.25 | 3.54 | 3.86 | 0.881 |
| Coriobacteriia | 3.70 | 4.93 | 4.68 | 1.70 | 3.23 | 4.79 | 0.881 |
| Fusobacteriia | 0.59 | 4.87 | 0.46 | 0.20 | 0.67 | 0.75 | 0.929 |
| Desulfovibrionia | 0.05 | 0.19 | 0.17 | 0.22 | 0.25 | 0.22 | 0.881 |
| unclassified_k__norank_d__Bacteria | 0.08 | 0.09 | 0.05 | 0.13 | 0.11 | 0.09 | 0.881 |
| unclassified_p__Firmicutes | 0.04 | 0.05 | 0.06 | 0.06 | 0.08 | 0.10 | 0.944 |

TableS2：Mean relative abundance (%) and *P* of the Order

| Species name | Pre | D1 | D3 | D5 | D7 | Follow-up visit | *P* |
| --- | --- | --- | --- | --- | --- | --- | --- |
| Lachnospirales | 38.76 | 39.88 | 47.79 | 33.68 | 41.74 | 37.65 | 0.901 |
| Oscillospirales | 13.29 | 13.55 | 12.47 | 11.84 | 12.76 | 15.20 | 0.988 |
| Enterobacterales | 15.30 | 5.64 | 5.01 | 8.98 | 18.10 | 6.23 | 0.988 |
| Bacteroidales | 7.77 | 3.36 | 4.25 | 13.52 | 9.64 | 8.33 | 0.901 |
| Bifidobacteriales | 8.31 | 8.59 | 12.02 | 8.37 | 1.42 | 7.11 | 0.901 |
| Veillonellales-Selenomonadales | 3.50 | 8.56 | 3.12 | 6.21 | 3.12 | 12.21 | 0.988 |
| Coriobacteriales | 3.70 | 4.93 | 4.68 | 1.70 | 3.23 | 4.79 | 0.901 |
| Peptostreptococcales-Tissierellales | 0.70 | 3.32 | 4.65 | 2.23 | 2.66 | 0.95 | 0.839 |
| Lactobacillales | 2.95 | 3.61 | 0.93 | 1.18 | 1.55 | 1.90 | 0.932 |
| Erysipelotrichales | 2.41 | 1.34 | 2.09 | 0.87 | 1.86 | 1.87 | 0.901 |

TableS3：Mean relative abundance (%) and *P* of the Family

| Species name | Pre | D1 | D3 | D5 | D7 | Follow-up visit | *P* |
| --- | --- | --- | --- | --- | --- | --- | --- |
| Lachnospiraceae | 38.76 | 39.88 | 47.79 | 33.68 | 41.74 | 37.65 | 0.877 |
| Ruminococcaceae | 12.46 | 11.88 | 11.12 | 9.20 | 10.13 | 12.62 | 0.973 |
| Enterobacteriaceae | 15.30 | 5.60 | 5.01 | 8.98 | 18.10 | 6.18 | 0.973 |
| Bifidobacteriaceae | 8.31 | 8.59 | 12.02 | 8.37 | 1.42 | 7.11 | 0.877 |
| Bacteroidaceae | 3.55 | 2.15 | 1.65 | 7.66 | 4.23 | 5.87 | 0.877 |
| Selenomonadaceae | 1.21 | 6.58 | 1.64 | 4.26 | 1.59 | 7.74 | 0.976 |
| Coriobacteriaceae | 3.54 | 4.40 | 4.18 | 1.54 | 2.90 | 4.41 | 0.889 |
| Prevotellaceae | 3.58 | 0.30 | 1.42 | 4.60 | 3.58 | 1.07 | 0.973 |
| Peptostreptococcaceae | 0.63 | 3.20 | 4.56 | 2.15 | 2.52 | 0.85 | 0.877 |
| Veillonellaceae | 2.30 | 1.98 | 1.48 | 1.95 | 1.53 | 4.47 | 0.973 |

TableS4：Mean relative abundance (%) and *P* of the Genus

| Genus | Pre | D1 | D3 | D5 | D7 | Follow-up visit | *P* |
| --- | --- | --- | --- | --- | --- | --- | --- |
| *Blautia* | 17.18 | 17.84 | 23.91 | 16.57 | 15.62 | 14.99 | 0.975 |
| *Faecalibacterium* | 9.44 | 8.08 | 7.01 | 6.23 | 7.34 | 7.97 | 0.998 |
| *Bifidobacterium* | 8.31 | 8.59 | 12.02 | 8.37 | 1.42 | 7.11 | 0.975 |
| *Escherichia-Shigella* | 14.99 | 3.74 | 3.12 | 8.26 | 4.50 | 5.44 | 0.980 |
| *Ruminococcus_torques_group* | 7.31 | 5.51 | 5.88 | 3.52 | 7.12 | 4.39 | 0.975 |
| *Bacteroides* | 3.55 | 2.15 | 1.65 | 7.66 | 4.23 | 5.87 | 0.975 |
| *Megamonas* | 1.21 | 6.58 | 1.64 | 4.26 | 1.59 | 7.74 | 0.998 |
| *Collinsella* | 3.48 | 4.39 | 4.16 | 1.53 | 2.90 | 4.40 | 0.975 |
| unclassified_f__Lachnospiraceae | 2.38 | 2.64 | 3.46 | 3.05 | 3.77 | 3.80 | 0.980 |
| unclassified_f__Enterobacteriaceae | 0.30 | 1.69 | 1.81 | 0.69 | 13.36 | 0.70 | 0.273 |
| *Agathobacter* | 2.07 | 2.82 | 3.53 | 2.43 | 2.48 | 3.41 | 0.998 |
| *Dorea* | 2.58 | 1.70 | 3.25 | 1.16 | 3.47 | 2.44 | 0.975 |
| *Prevotella* | 3.55 | 0.25 | 1.37 | 4.59 | 3.49 | 0.95 | 0.988 |
| *Eubacterium_hallii_group* | 1.67 | 2.95 | 3.08 | 1.76 | 2.18 | 2.26 | 0.975 |
| *Romboutsia* | 0.53 | 3.06 | 4.49 | 1.97 | 2.37 | 0.75 | 0.975 |
| *Subdoligranulum* | 1.83 | 2.23 | 2.68 | 2.01 | 1.74 | 1.31 | 0.998 |
| *Clostridium_sensu_stricto_1* | 0.36 | 0.36 | 0.09 | 6.91 | 0.09 | 0.01 | 0.975 |
| *Cetobacterium* | 0.56 | 4.86 | 0.44 | 0.19 | 0.64 | 0.50 | 0.998 |
| *Ruminococcus* | 0.83 | 1.15 | 1.19 | 0.53 | 0.54 | 2.89 | 0.998 |
| *Dialister* | 0.48 | 1.68 | 1.09 | 1.48 | 0.98 | 0.69 | 0.998 |
| *Megasphaera* | 1.43 | 0.18 | 0.33 | 0.25 | 0.36 | 3.66 | 0.998 |
| *Anaerostipes* | 0.99 | 1.61 | 0.62 | 0.85 | 0.80 | 0.76 | 0.975 |
| *Streptococcus* | 2.44 | 1.27 | 0.49 | 0.78 | 0.32 | 0.23 | 0.975 |
| *Phascolarctobacterium* | 1.36 | 0.34 | 0.53 | 1.64 | 0.62 | 0.63 | 0.998 |
| *UCG-002* | 0.10 | 0.46 | 0.47 | 1.33 | 1.24 | 1.48 | 0.980 |

TableS5：Significantly different species at each taxonomic level

(Prefix letters represent phylum, class,order, family, and genus)

| Species name | Pre | D1 | D3 | D5 | D7 | Follow-up visit | *P* |
| --- | --- | --- | --- | --- | --- | --- | --- |
| p_Cyanobacteria | 0 | 0.003558 | 0.008451 | 0.002891 | 0 | 0.000222 | 0.0029 |
| c_Cyanobacteriia | 0 | 0 | 0.003113 | 0 | 0 | 0 | 0.0001 |
| o_Bacillales | 0 | 0.003336 | 0.004003 | 0 | 0 | 0.000222 | 0.0195 |
| o_Chloroplast | 0 | 0 | 0.003113 | 0 | 0 | 0 | 0.0003 |
| f_Leuconostocaceae | 0.1472 | 0.1032 | 0.002224 | 0.006227 | 0.3087 | 0.003336 | 0.0176 |
| f_Bacillaceae | 0 | 0.003336 | 0.004003 | 0 | 0 | 0.000222 | 0.0255 |
| f_norank_o__Chloroplast | 0 | 0 | 0.003113 | 0 | 0 | 0 | 0.0006 |
| *g_Weissella* | 0.002446 | 0.103 | 0.002001 | 0.006227 | 0.29 | 0.1463 | 0.0174 |
| *g_Phocea* | 0 | 0.005337 | 0.000445 | 0.002891 | 0.01534 | 0.03069 | 0.0174 |
| *g_norank_f__norank_o__Chloroplast* | 0 | 0 | 0.003113 | 0 | 0 | 0 | 0.0015 |

TableS6: Stability of Microbiome Phenotypes Pre- and Post-TRQ Treatment as Analyzed by BugBase (Mean abundance)

| Phenotypes | Pre | D1 | D3 | D5 | D7 | Follow-up visit | *P* |
| --- | --- | --- | --- | --- | --- | --- | --- |
| Anaerobic | 34.01 | 36.99 | 38.33 | 37.13 | 31.56 | 35.95 | 0.948 |
| Gram_Positive | 32.76 | 35.92 | 37.86 | 33.98 | 29.98 | 32.76 | 0.948 |
| Potentially_Pathogenic | 9.29 | 7.84 | 5.94 | 9.59 | 9.74 | 10.62 | 0.795 |
| Contains_Mobile_Elements | 6.37 | 6.71 | 6.51 | 5.07 | 7.85 | 5.89 | 0.948 |
| Forms_Biofilms | 6.73 | 6.14 | 6.45 | 5.11 | 6.03 | 6.02 | 0.948 |
| Gram_Negative | 4.97 | 3.42 | 2.34 | 5.76 | 6.53 | 5.69 | 0.471 |
| Facultatively_Anaerobic | 3.26 | 1.63 | 1.46 | 1.72 | 4.20 | 1.65 | 0.948 |
| Stress_Tolerant | 2.47 | 1.29 | 1.03 | 1.49 | 3.96 | 1.30 | 0.948 |
| Aerobic | 0.13 | 0.07 | 0.08 | 0.14 | 0.18 | 0.12 | 0.471 |
